# Development of a Click-Chemistry Reagent Compatible with Mass Cytometry

**DOI:** 10.1038/s41598-018-25000-y

**Published:** 2018-04-27

**Authors:** Jessica Shaklee, Kriti Srivastava, Heather Brown, Edgar A. Arriaga, Valerie C. Pierre, Jop H. van Berlo

**Affiliations:** 1 Department of Medicine, Cardiovascular Division, University of Minnesota, Minneapolis, MN United States; 2 Department of Chemistry, University of Minnesota, Minneapolis, MN United States; 3 Lillehei Heart Institute, University of Minnesota, Minneapolis, MN United States; 4 Stem Cell Institute, University of Minnesota, Minneapolis, MN United States

## Abstract

The recent development of mass cytometry has allowed simultaneous detection of 40 or more unique parameters from individual single cells. While similar to flow cytometry, which is based on detection of fluorophores, one key distinguishing feature of mass cytometry is the detection of atomic masses of lanthanides by mass spectrometry in a mass cytometer. Its superior mass resolution results in lack of signal overlap, thereby allowing multiparametric detection of molecular features in each single cell greater than that of flow cytometry, which is limited to 20 parameters. Unfortunately, most detection in mass cytometry relies on lanthanide-tagged antibodies, which is ideal to detect proteins, but not other types of molecular features. To further expand the repertoire of molecular features that are detectable by mass cytometry, we developed a lanthanide-chelated, azide-containing probe that allows click-chemistry mediated labeling of target molecules. Following incorporation of the thymidine analog 5-ethynyl-2′-deoxyuridine (EdU) during DNA synthesis in S-phase of the cell cycle, we demonstrate that the probe introduced here, tagged with Terbium-159 (^159^Tb), reacts via copper-catalyzed azide-alkyne Huisgen cycloaddition (click-chemistry) with Edu. Thus, detection of ^159^Tb makes it possible to measure DNA synthesis in single cells using mass cytometry. The approach introduced here shows similar sensitivity (true positive rate) to other methods used to measure DNA synthesis in single cells by mass cytometry and is compatible with the parallel antibody-based detection of other parameters in single cells. Due to its universal nature, the use of click-chemistry in mass cytometry expands the types of molecular targets that can be monitored by mass cytometry.

## Introduction

Flow cytometry has been critical to advance our understanding of the immune system as well as other complex biological systems^1^. Flow cytometry is the method of choice to detect up to 20 molecular targets in parallel in or on individual cells, to define cellular subtypes, and to generate population statistics^2^. However, despite the development of new reagents, the inherent broad fluorescence emission spectrum of each fluorophore used in flow cytometry results in spectral overlap with other fluorophore emission spectra, requiring mathematical compensation to resolve separate emission spectra and assign fluorescent signals to each molecular target^2–5^. Similar to flow cytometry, mass cytometry can measure molecular targets in and on individual cells. But in contrast to fluorescence detection, mass cytometry uses detection of atomic masses, which can be resolved at unit mass resolution with less than 0.1% overlap, obviating the need for compensation to resolve spectral overlap^1,6^. Similar to flow cytometry, mass cytometry is based on the use of antibodies, but for mass cytometry these are tagged with isotopically pure lanthanide metal ions, which are typically absent in living cells^6–8^. In the mass cytometer, as nebulization and atomization of each individual cell proceed sequentially, the higher-mass ions, including lanthanide ions, are selected and detected^7,8^. Thus, the detection of lanthanide ions in a particular cell corresponds to the presence of the tagged antibody and molecular target pair in that cell prior to nebulization and atomization since no cellular molecules contain lanthanides^6–8^.

Multiparametric detection by mass cytometry has many applications and has made it possible to identify populations of immune cells with higher precision, characterize drug responses in multiple cell types simultaneously, and describe dynamics of cell differentiation^9,10^. Mass cytometry has also been used to monitor DNA synthesis^11^. Incorporation of 5-iodo-2′-deoxyuridine (IdU) during DNA synthesis can be monitored by mass cytometry because iodine-127 is isotopically stable and falls within the measurable mass range of mass cytometry^12^. This technique works well for short-term pulsing in cell culture, where IdU is added for the last couple of minutes before harvesting cells. Unfortunately, IdU has inhibitory effects on cellular proliferation that could compromise long-term tracking of cellular renewal during *in vivo* experiments^12–15^. Thymidine analog 5-bromo-2′-deoxyuridine (BrdU) does not have inhibitory effects on cellular proliferation and detection of BrdU incorporated during DNA synthesis is achievable by mass cytometry using anti-Brdu antibodies tagged with lanthanide metal ions. The drawback of antibody-based BrdU detection is that all available antibodies recognize incorporated BrdU only on single-stranded DNA^16^. Therefore detection of incorporated BrdU requires harsh treatments with acid or DNAse to allow denaturation of the DNA, which could interfere with antibody labeling and detection of other proteins that are planned in multi-antibody stainings^2,17^. An alternative to BrdU that is detectable without denaturing DNA has been developed for immunocytochemistry and flow cytometry^11,18^. This alternative uses incorporation of 5-ethynyl-2′-deoxyuridine (EdU) followed by copper-catalyzed azide-alkyne Huisgen cycloaddition (click-chemistry) with a fluorescent probe to detect EdU^11,19,20^. However, there is currently no click-chemistry reagent commercially available that is compatible with mass cytometry, i.e. a lanthanide-tagged probe. Although a recent publication laid out one possible strategy to detect incorporated EdU with mass cytometry, we propose a more versatile strategy that allows labeling with a lanthanide ion of choice^21^.

## Results

**Figure 1:**
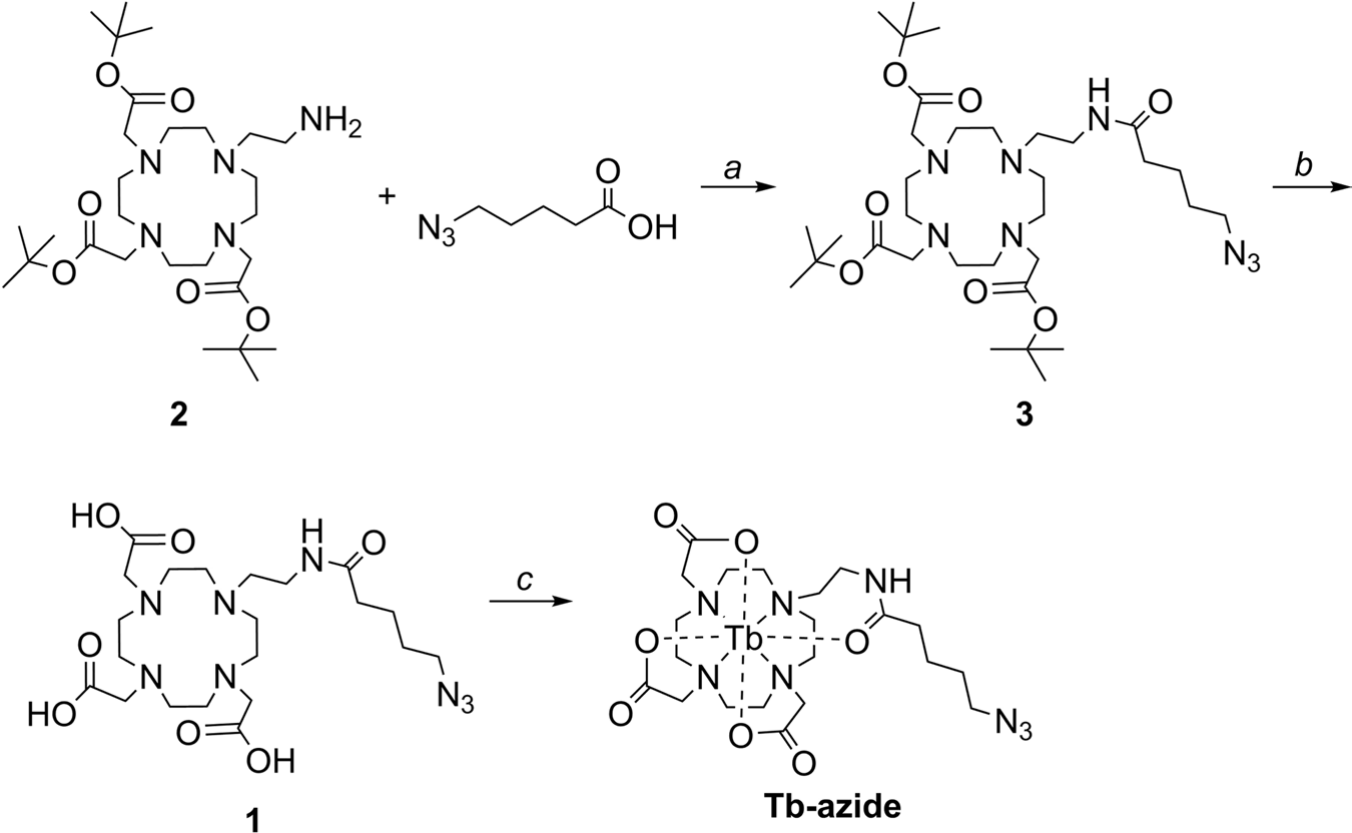
Synthetic scheme for Tb-azide. Ligand 2,2′2″-(10-(2-(5-azidopentanamido)ethyl)-1,4,7,10-tetraazacyclododecane-1,4,7-triyl)triacetic acid (DOTA-Et-pentanamidoazide) (**1**) was synthesized in two steps starting from precursor tri-*tert*-butyl 2,2′,2″-(10-(2-aminoethyl)-1,4,7,10-tetraazacyclododecane-1,4,7-triyl)triacetate (DO3A-Et-amine) (**2**). The precursor **2** was synthesized in seven steps following the reported literature procedure^45,46^. Coupling of precursor **2** with 5-azidopentanoic acid by using peptide coupling reagent 1-[bis(dimethylamino)methylele]-1*H*-1,2,3-triazol[4,5-*b*]pyridinium 3-oxid hexafluorophosphate (HATU) and N, N-diisopropylethylamine (DIPEA) in solvent dry N, N-dimethylformamide (DMF) at room temperature (RT) gave tri-*tert*-butyl 2,2′,2″-(10-(2-(5-azidopentanamido)ethyl)-1,4,7,10-tetraazacyclododecane-1,4,7-triyl)triacetate (DO3A-Et-pentanamidoazide) (**3**). Deprotection of the *tert*-butyl groups with trifluoroacetic acid (TFA) in CH_2_Cl_2_ (Dichloromethane) at 0 °C yielded the deprotected ligand **1**. The probe Tb-azide was synthesized by heating an equimolar quantity of the ligand **1** and TbCl_3_∙6H_2_O in CH_3_OH and H_2_O (1:1) mixture at 70 °C for 3 days at neutral pH. Reagents and conditions: (**a**) DMF, HATU, DIPEA, 0 °C-RT, 40 h, 96%; (**b**) Dichloromethane, TFA, 0 °C-RT, 24 h, quantitative; (**c**) TbCl_3_^·^6H_2_O, NaOH(aq.), H_2_O: MeOH (1:1), pH~7, 70 °C, 72 h, 96%.

Labeling reagents that are based on click-chemistry have demonstrated versatility in multiple types of assays (e.g. fluorescence and mass spectrometry)^11,22–24^. To demonstrate feasibility of a generic click-chemistry approach that results in labeling of molecular targets with lanthanides that are commonly used in mass cytometry analyses, we detected by mass cytometry the incorporation of EdU, a thymidine analog, into DNA during DNA replication in currently or previously proliferating cells (Fig. 1 and Methods). EdU is a commonly used reagent that is easily introduced to proliferating cells, and its detection by immunocytochemistry and flow cytometry are well established, and thus could serve as a platform to validate our novel method of labeling and detecting EdU by lanthanide probe and mass cytometry^11,17,25^. For fluorescence microscopy the click-chemistry reaction between an azide-containing fluorophore and EdU molecules that had been incorporated into the DNA during the replication phase of cell cycle was confirmed through colocalization with the DNA counterstain 4′,6-Diamidino-2-Phenylindole (DAPI, Supplementary Fig. 1)^26^. Fluorescence microscopy showed incorporation of BrdU and/or EdU in the nuclei of correspondingly stained cells, indicative of cells currently or previously in S phase.

Both flow and mass cytometry commonly use similar sample preparation procedures. Thus, monitoring the incorporation of EdU into the DNA of HeLa cells by flow cytometry, provided further evidence that cell preparation and staining would likely be compatible with mass cytometry procedures. Similar to the fluorescence microscopy procedures, BrdU was detected using monoclonal anti-BrdU antibodies and Edu was detected via click-chemistry with an azide-containing fluorophore. DNA was counterstained with propidium iodide (PI). Based on non-pulsed cells that were similarly treated with click-chemistry and antibodies, we determined background signal and, by comparison, the threshold above which a signal is considered positive, indicative of the presence of BrdU or EdU. We readily detected BrdU, EdU or both combined incorporated into replicating DNA by flow cytometry (Supplementary Fig. 2), which confirms the feasibility of using HeLa cells and EdU as a system to test the generic click-chemistry approach for mass cytometry described below (Fig. 1).

**Figure 2:**
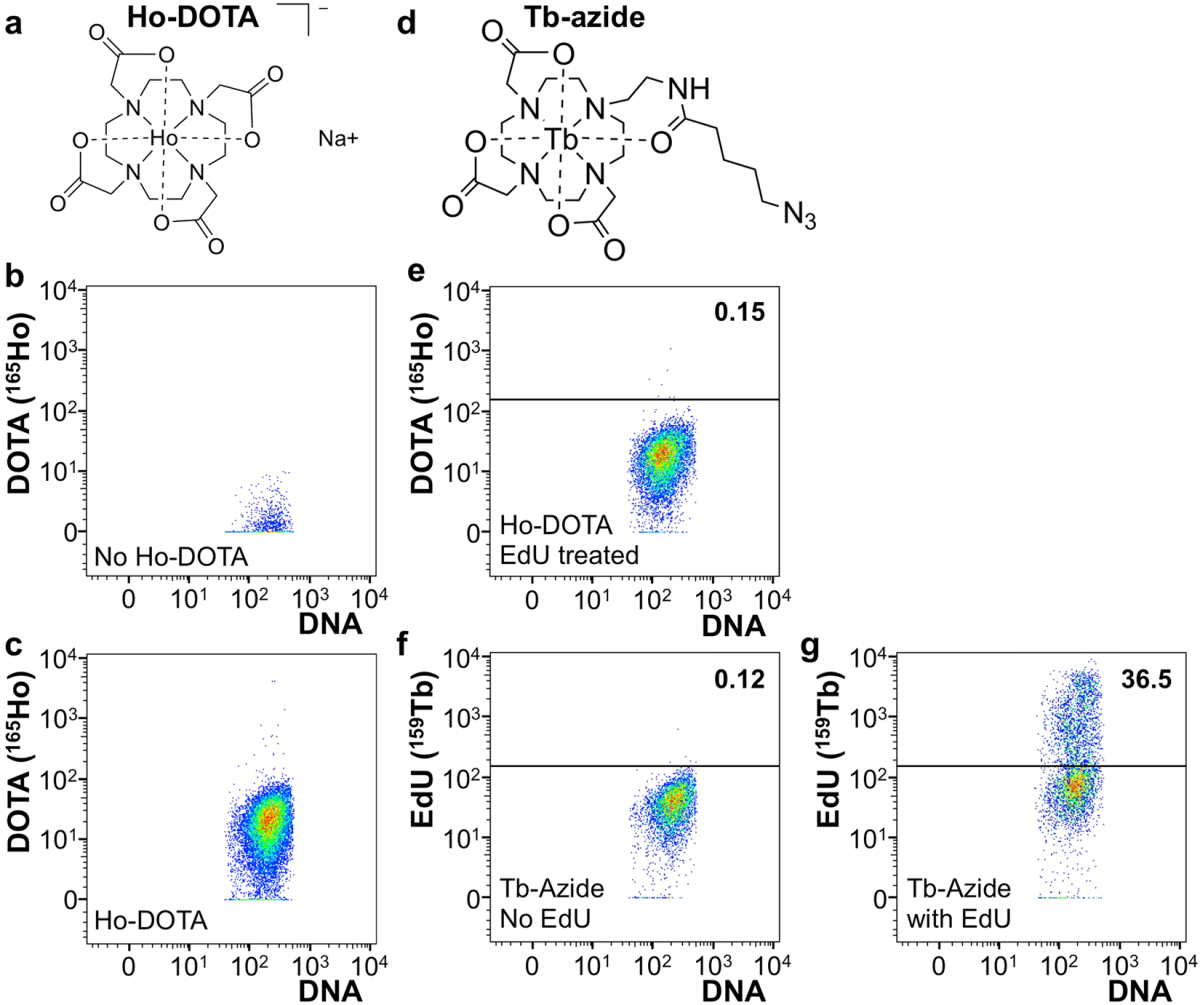
Structure and validation of click chemistry with mass cytometry. (**a**) Structure of DOTA, chelated with holmium (Ho-DOTA). (**b**) Mass cytometry plot of cells that were measured for background signals in the holmium channel (not stained with Ho-DOTA). DNA is used as a marker to detect cellular events, not to quantify DNA content. (**c**) Mass cytometry plot of cells that were stained with Ho-DOTA under click chemistry reaction conditions. (**d**) Structure of azide containing DOTA, chelated with terbium (Tb-azide). (**e**) Mass cytometry plot of cells that were treated with EdU and stained with Ho-DOTA under click chemistry reaction conditions. (**f**) Mass cytometry plot of cells that were not treated with EdU and stained with Tb-azide under click chemistry reaction conditions. (**g**) Mass cytometry plot of cells that were treated with EdU and stained with Tb-azide under click chemistry reaction conditions. (**e–g**) Number above line shows percentage of cells considered positive for respective channel for the sample shown. DNA stain is used to detect cellular events.

The compound 1,4,7,10-tetraazacyclododecane-1,4,7,10-tetraacetic acid (DOTA) is known for its ability to chelate lanthanide ions^27–31^. We chelated ^165^Ho^3+^ with unmodified DOTA (Ho-DOTA) containing no azide group for use as a negative control reagent (Fig. 2a, Ho-DOTA). When cells were exposed to Ho-DOTA under conditions typical of click-chemistry reactions for biological systems, mass cytometry revealed a low increase in ^165^Ho background signal from this compound compared to non-exposed cells, likely indicative of low-level, non-specific binding of Ho-DOTA to fixed, permeabilized cells (Fig. 2b,c). In all mass cytometry experiments, cells were counterstained with DNA-Iridium-191, to allow identification of cellular events. When EdU-treated cells were exposed to Ho-DOTA in a click-chemistry reaction (as a negative control, since Ho-DOTA does not contain an azide moiety) we detected no enhanced ^165^Ho signal with mass cytometry compared to non-EdU treated cells (Fig. 2c,e). These negative controls, define a low-level, non-specific binding of Ho-DOTA to permeabilized cells.

The DOTA-based azide-containing probe (Fig. 2d**)** was synthesized as outlined in Fig. 1 and “Methods for Synthesis Scheme”, and chelated with ^159^Tb^3+^ to yield the desired probe (Tb-azide) (Figs 1 and 2d). When cells that were not treated with EdU were subjected to click-chemistry reaction conditions in the presence of Tb-azide, mass cytometry reported low levels of ^159^Tb. These results are similar to the negative controls of cells exposed to Ho-DOTA (Fig. 2f). All cells were clustered together as a single population, without any discernable ‘positive’ population, consistent with low-level, non-specific binding of Tb-azide to permeabilized cells. In contrast, cells treated with EdU and subjected to click-chemistry conditions in the presence of Tb-azide resulted in a distinctly positive population, indicating successful detection of incorporated EdU by mass cytometry (compare Fig. 2f,g).

**Figure 3:**
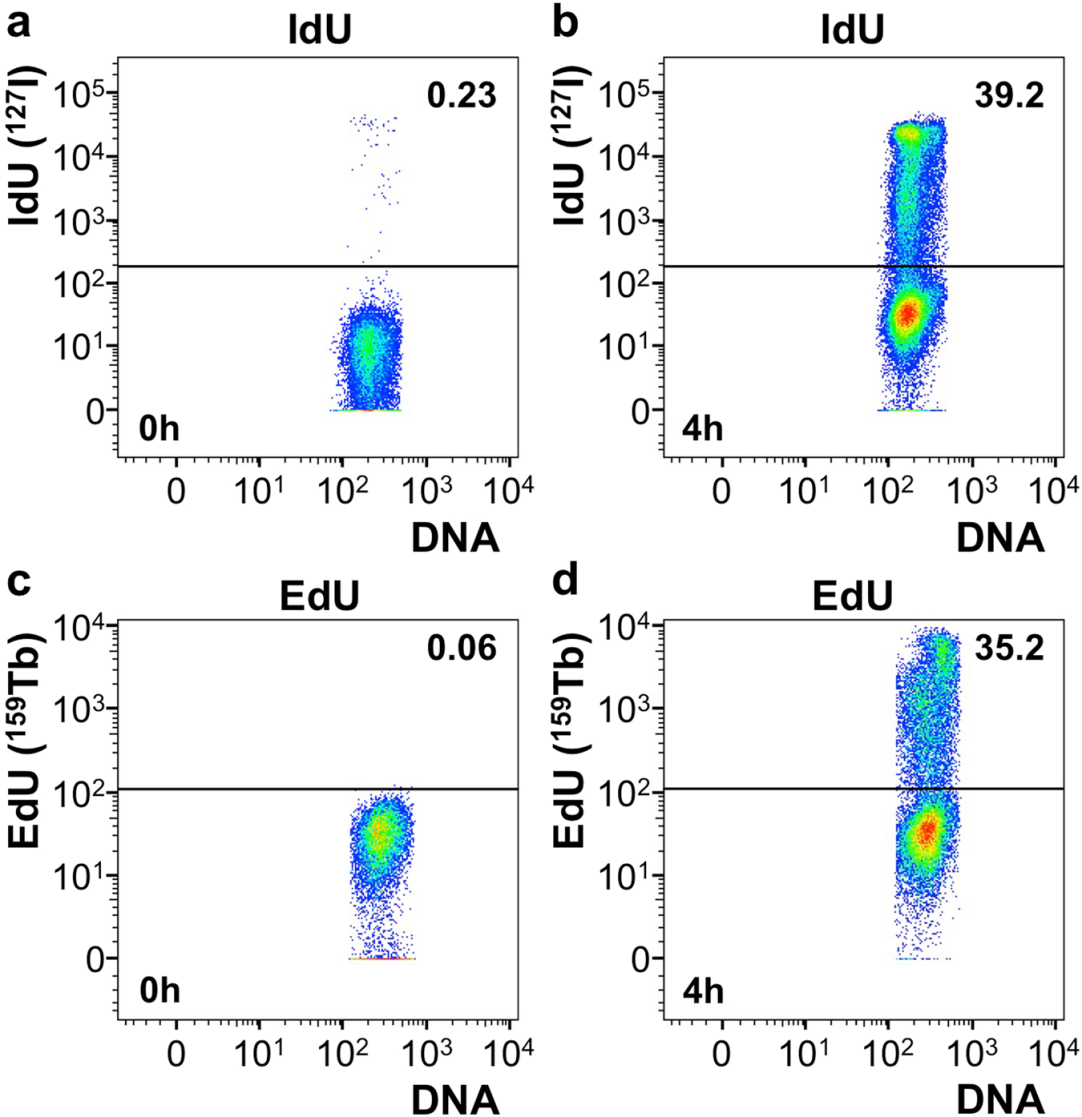
Comparison between IdU and EdU to detect DNA replication with mass cytometry. (**a**) Mass cytometry plot of cells that were not treated with IdU. (**b**) Mass cytometry plot of cells that were treated with IdU. (**c**) Mass cytometry plot of cells that were not treated with EdU and stained with Tb-azide. (**d**) Mass cytometry plot of cells that were treated with EdU and stained with Tb-azide. (**a–d**) Number in upper right corner shows percentage of cells that is positive for respective channel for samples shown. DNA stain is used to detect cellular events.

Because 5-iodo-2′-deoxyuridine incorporation into DNA is also used to monitor DNA synthesis by mass cytometry^12^, we next compared the ability to detect DNA-incorporated EdU by click-chemistry using Tb-azide with measurement of incorporated IdU (Fig. 3a–d). In either case, when cells were not treated with IdU or EdU, the level of false positive signal was low (0.64% vs 0.32% resp.). When cells were treated with IdU or EdU, and exposed to click-chemistry reaction conditions in the presence of Tb-azide, both methods showed distinctively positive populations (43.8% and 32.0%, respectively).

**Figure 4:**
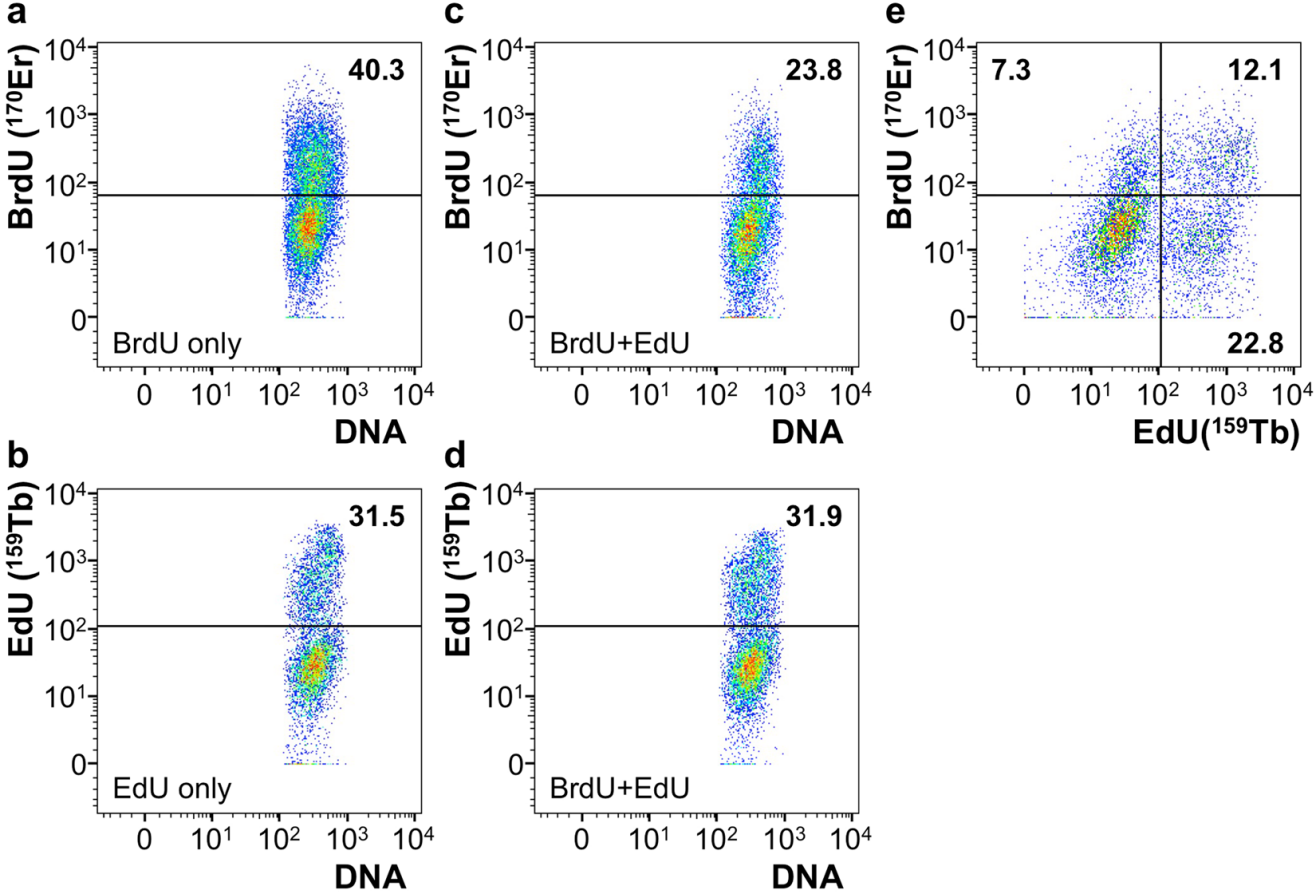
Comparison between BrdU and EdU to detect DNA replication with mass cytometry. (**a**) Mass cytometry plot of cells that were treated with BrdU and stained with BrdU antibody. (**b**) Mass cytometry plot of cells that were treated with EdU and stained with Tb-azide. (**c**) Mass cytometry plot showing BrdU vs DNA channel of cells that were treated with EdU and BrdU and stained with BrdU and Tb-azide. (**d**) Mass cytometry plot showing EdU vs DNA channel of cells that were treated with EdU and BrdU and stained with BrdU and Tb-azide. (**e**) Mass cytometry plot showing BrdU vs EdU channel of cells that were treated with EdU and BrdU and stained with BrdU and Tb-azide. (**a–e**) Numbers show percentage of cells that is positive for respective channel for the samples shown. DNA stain is used to detect cellular events.

To further analyze the ability of Tb-azide to detect incorporated EdU with mass cytometry, we compared measurement of EdU incorporation detected by Tb-azide with measurement of BrdU incorporation detected by Erbium-170 (^170^Er^3+^) conjugated monoclonal anti-BrdU antibodies. Incorporation of BrdU or EdU into DNA resulted in detection of ^170^Er or ^159^Tb signal in individual cells, respectively (Fig. 4a,b). False positive rates were comparable, although the BrdU antibody labeled a small proportion of cells that had only EdU (and no BrdU) incorporated, due to minimal cross-reactivity of anti-BrdU antibodies (data not shown). Incorporation of both EdU and BrdU resulted in detection of both EdU and BrdU in individual cells (Fig. 4c–e). However, levels of BrdU detection were reduced for unknown reasons. We had previously observed a similar drop in levels of BrdU detection for double-incorporation experiments detected with flow cytometry (data not shown), so the phenomenon may be due to preferential incorporation of the various thymidine analogs, or potentially due to competition between the 2 detection methods. It could be that detection of BrdU is prevented by previous click-chemistry labeling of neighboring incorporated EdU.

**Figure 5:**
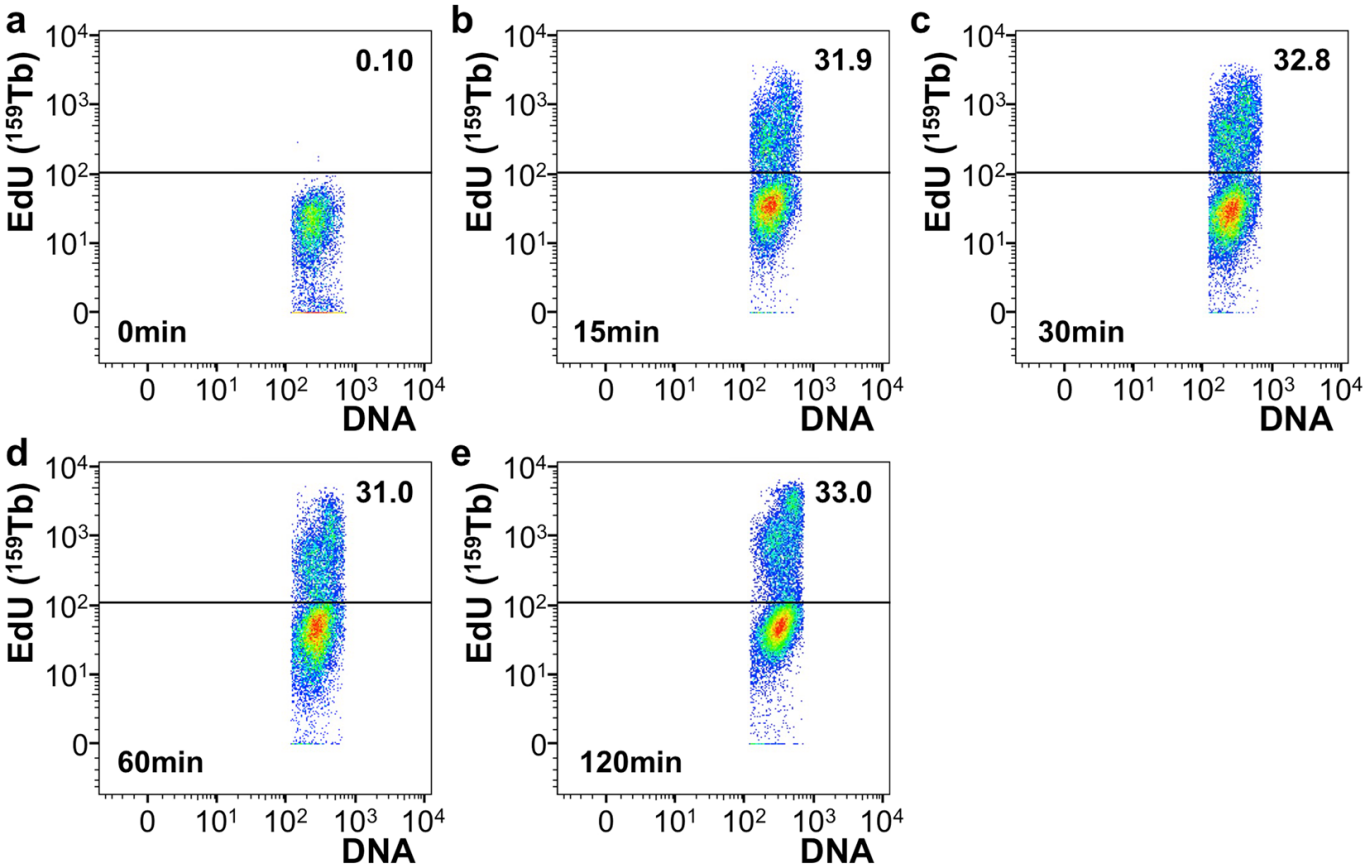
Time course of EdU incorporation detected with mass cytometry. (**a**) Mass cytometry plot showing EdU vs DNA channel of cells that were not treated with EdU (**b**) Mass cytometry plot showing EdU vs DNA channel of cells that were treated with EdU for 15 min. (**c**) Mass cytometry plot showing EdU vs DNA channel of cells that were treated with EdU for 30 min. (**d**) Mass cytometry plot showing EdU vs DNA channel of cells that were treated with EdU for 60 min. (**e**) Mass cytometry plot showing EdU vs DNA channel of cells that were treated with EdU for 120 min. Numbers show percentage of cells that is positive for EdU for the samples shown. DNA stain is used to detect cellular events.

Finally, we conducted a time-course study to assess the sensitivity of detection of EdU incorporation into DNA, based on the click-chemistry reaction with Tb-azide. Cells were pulsed with EdU for up to 120 minutes. Surprisingly, the fraction of cells that incorporated EdU reached a plateau already within 15 minutes of EdU exposure (Fig. 5a–e). These findings are in agreement with the detection of IdU to measure cell cycle with mass cytometry^12^, and are likely indicative of the fraction of cells in S-phase of the cell cycle in an unsynchronized HeLa cell culture at any given time.

## Discussion

Here, we described a versatile approach for mass cytometry based detection of individual functional cell properties in individual cells based on the reaction of a lanthanide-chelated probe (Tb-azide) via click-chemistry reaction with EdU. Immediately, this approach would be highly valuable for long-term tracing of EdU incorporation, which results in minimal toxicity for identification of cellular progeny^13,18,32,33^. Furthermore, the mass cytometry probe could be easily adapted to other applications that use click-chemistry compatible reporters^11,34^.

DOTA was chosen as the basis of the mass cytometric probe because of its ability to chelate lanthanide ions, such as gadolinium^27–31,35^. The probe was designed to contain an azide group, a choice driven by the frequent use of alkyne-containing reagents in biological systems, such as EdU. On the other hand, it would be relatively straightforward to design DOTA with an alkyne, or any other click-chemistry compatible functional group instead of an azide^36^. Thus, this strategy is highly customizable to various targets in various experimental contexts.

The sensitivity of the Tb-azide probe for detection of incorporation of EdU into DNA is comparable to either IdU, which is detected directly by the mass cytometer, or to BrdU, which is detected by BrdU antibody that was conjugated to ^170^Er. In contrast to BrdU, EdU detection of DNA synthesis does not require strong acid treatments, which could interfere with antibody-based detection of other proteins.

Given the abundance of EdU incorporated into DNA, even after 15 minutes of exposure to EdU, the mass cytometry detection thresholds were readily met (Fig. 5b). However, monitoring other cellular events that result in lower levels of incorporated alkynes in single cells, might require further modifications to the DOTA-based reagent to bring the chelated ion content above detection thresholds by the mass cytometer^7^. Indeed, novel strategies to increase the efficiency of click-chemistry labeling (e.g. by allowing locally chelated Cu^+^ to catalyze and enhance the cycloaddition reaction^37^), could further enhance the detection capabilities of the probe introduced here. This could lead to the development of additional applications for our lanthanide-chelated probe in detecting non-protein events, such as lipid reactions, by mass cytometry, similar to click-chemistry compatible assays available for flow cytometry^34,38,39^.

Analysis of the cell cycle by flow cytometry has a long history, and the use of click chemistry to analyze DNA replication as well as other cellular events such as RNA synthesis has made this much easier to combine with multi-parameter panels^39^. Flow cytometry is clearly the technique of choice to analyze cell cycle kinetics. However, when studying more complex biological systems, and where more than 20 parameters are needed, mass cytometry offers clear advantages, despite the required software to analyze these high-dimensionality datasets^40–42^. Given the known cytotoxic effects of IdU on cellular proliferation, and the widespread use of EdU for labeling proliferative cells within tissues, the Tb-azide probe provides a valuable solution to monitor DNA synthesis and cellular renewal in tissues by mass cytometry^11,14^. Such studies could be extended (e.g. by using barcoding techniques), to map cellular progenies of progenitor cells across multiple generations^10,43^.

Although another approach to allow detection of incorporated EdU with mass cytometry was recently published, we believe our approach to be more versatile^21^. This former approach did not use an azide-containing probe that can be loaded with any lanthanide ion and can be directly detected with mass cytometry, but instead used a 5-iodo-5′-azido-2′,5′-dideoxyuridine that contained an iodine molecule to perform click-chemistry^21^. In comparison, our approach is more generic and adaptable, and uses an azide-containing probe that can be loaded with any lanthanide ion of choice. Moreover, we provide detailed instructions and methodology on the generation of this probe.

In conclusion, Tb-azide is a mass cytometric probe, which demonstrates the feasibility of using click-chemistry to detect functional alkyne-containing probes in single cells. This probe is not dependent on antibodies and can be used with a multitude of lanthanide tags, allowing for simultaneous, multi-parametric detection of molecular targets via antibodies with RNA synthesis, lipid regulation of proteins and other emerging applications in the field of mass cytometry.

## Methods

### Cell Culture

HeLa cells (purchased from ATCC) were cultured in DMEM supplemented with 10% Bovine Growth Serum, 1% L-glutamine and 0.5% Pen-Strep. Cells were passaged with Trypsin, and plated in 15 cm diameter plates for flow and mass cytometry experiments. Thymidine analogs BrdU, EdU or IdU were added at 10 µM at indicated times before cells were harvested. For double incorporation of BrdU and Edu, cells were first pulsed with EdU for 4 hr, followed by BrdU for an additional 4 hr, due to the preferential incorporation of BrdU when both EdU and BrdU are present^44^. Cell Culture experiments were carried out in accordance with the relevant guidelines and approved by the University of Minnesota Institutional Biosafety Committee.

### Cell staining

Cells were fixed with 100% EtOH in 12-well plates for immunocytochemistry or in solution for cytometry experiments. Cells were washed in PBS, followed by incubation in 3–4 N HCl for 20 min at RT. After acid treatment cells were stained with click-chemistry cocktail following manufacturer’s protocol (Thermo Fisher C10337) for 30 min at RT and protected from light, although click-chemistry using the following cocktail also worked (100 mM Tris.HCl pH 8.5, 4 mM CuSO_4_, 10 µM azide-group containing reagent, 100 mM L-Ascorbate). For mass cytometry, we substituted the fluorophore-conjugated azide with Tb-azide at 10 µM. After performing click-chemistry mediated labeling of EdU, cells were washed and further stained with BrdU antibodies (MoBu-1 for flow cytometry, and ^170^Er^3+^-conjugated MoBu1 for mass cytometry), followed by additional washes. For flow cytometry, cells were further treated with fluorophore-conjugated secondary antibody (Life Technologies) and propidium iodide (PI, 50 µg/mL) and RNAse (200 µg/mL) to stain DNA. For mass cytometry cells were stained overnight at 4 °C with the Cell-ID Intercalator-Ir (Fluidigm) in MaxPar fix-and-perm buffer (Fluidigm) to allow detection of cellular events with mass cytometry.

### Antibodies

BrdU was detected by MoBu-1 (Biolegend), although other BrdU antibodies were used in pilot experiments to determine cross-reactivity with EdU. Since antibody-based detection of incorporated BrdU could show cross-reactivity with incorporated EdU, we tested different monoclonal BrdU antibodies to identify which antibody was most specific to BrdU with minimal cross-reactivity to EdU. Similar to published data, we found that the monoclonal antibody MoBu1 showed the least cross-reactivity with EdU among 3 different antibodies tested (BU-33, BU-1 and MoBu-1, data not shown)^17^.

### Antibody labeling

Kit provided ionic Erbium 170 was conjugated to MoBu-1 antibody using a conjugation kit following the manufacturer’s protocol (Maxpar Antibody Labeling Kit, Fluidigm). The yield of conjugated ^170^Er-MoBu-1 antibody was determined using a spectrophotometer.

### Flow cytometry

Stained samples were analyzed on a FACS Aria II flow cytometer (BD Biosciences). The flow cytometer is equipped with 488, 561 and 647 lasers with appropriate filters and PMTs for detection. Results were analyzed using FlowJo software. Each sample was gated for single cells according to cell size and ratio, and background signal was determined by comparison with negative controls. Negative controls included staining in the absence of antigen (such as BrdU antibody staining on cells that were not pulsed with BrdU), and no antibody controls. Gating of positive signal was based on negative control staining in absence of antigen.

### Mass cytometry

Stained cell samples were centrifuged (500 × g, 5 min), and the excess Intercalator-Ir was removed in the supernatant. The cells were resuspended in Maxpar cell staining buffer (Fluidigm) and centrifuged twice to remove all salts. Aliquots of each sample were applied to a hemocytometer to determine cell counts. The samples were washed once in MilliQ H_2_O and centrifuged, and the supernatant was removed as completely as possible. Pellets were then kept on ice until immediately prior to analysis.

Calibration bead solution (Fluidigm) was run on the CyTOF2 mass cytometer (Fluidigm) to calibrate it. Each sample was resuspended in a 1:10 dilution of the bead solution to a concentration of 2.5–5.0 × 10^5^ cells/mL and filtered immediately before injection into the instrument. The instrument was thoroughly washed with deionized water between each sample. Results were analyzed using FlowJo software. Each sample was gated for single cells according to DNA content (DNA-Ir), and bead-cell doublets were removed. Background signal was determined by comparison with negative controls. Negative controls included staining in the absence of antigen (such as BrdU antibody staining on cells that were not pulsed with BrdU), and no antibody controls. Gating of positive signal was based on negative control staining in absence of antigen.

### Statistics

Differences between groups were assessed by Student’s t-test. A p value < 0.05 was considered significant.

### Methods for Synthesis Scheme

#### General Considerations

Starting materials were obtained from commercial suppliers and were used without further purification. Ho-DOTA complex was synthesized following the reported literature procedure with successful synthesis confirmed by MS and ^1^H NMR^28^. Water was distilled and further purified by a Millipore cartridge system (resistivity 1.8 × 10^7^ Ω.cm). ^1^H spectra were obtained at room temperature on Varian Inova 500 at 500 MHz. ^13^C NMR spectra were obtained on Varian Inova 500 and Bruker Avance III HD 500 at 125.7 MHz at the LeClaire-Dow Characterization Facility of the Department of Chemistry at the University of Minnesota. Data for ^1^H NMR is reported as follows: chemical shift (*δ*, ppm), multiplicity (br = broad, s = singlet, d = doublet, t = triplet, m = multiplet, qunit = quintet), coupling constants (Hz). A delay time of 30 ms and acquisition time of 64 ms was used for the collection of ^1^H NMR spectra of the paramagnetic Tb-azide and Ho-DOTA complexes. The residual solvent peak was used as an internal reference for ^1^H NMR. Mass spectra (HRMS, high resolution mass spectrometry, and ESI-MS, electrospray ionization mass spectrometry) were recorded on a Bruker BioTOF II at the Waters Center for Innovation in Mass Spectrometry of the Department of Chemistry at the University of Minnesota.

Tri-*tert-butyl 2,2′,2″(10-(2-(5-azidopentanamido)ethyl)-1,4,7,10-tetraazacyclododecane-1,4,7-triyl)triacetate* (Fig. 1, 3). A solution of 5-azidopentanoic acid (15.4 mg, 0.107 mmol), tri-*tert*-butyl 2,2′,2*″*-(10-(2-aminoethyl)-1,4,7,10-tetraazacyclododecane-1,4,7-triyl)triacetate (Fig. 1, 2, 40 mg, 0.072 mmol) and N, N-diisopropylethylamine (DIPEA, 26.5 μL, 0.152 mmol) in anhydrous N, N-dimethylformamide (DMF, 3 mL) were stirred for 5 min at RT. The reaction mixture was then cooled to 0 °C, followed by the addition of 1-[Bis(dimethylamino)methylene]-1*H*-1,2,3-triazolo[4,5-b]pyridinium 3-oxid hexafluorophosphate (HATU, 29 mg, 0.075 mmol) and subsequently stirred for 10 min at 0 °C, and then for 40 h at RT. The reaction mixture was diluted with H_2_O (2 mL) and extracted with ethyl acetate (5 × 4 mL). The combined organic extracts were dried over MgSO_4_ (*s*) and concentrated under reduced pressure. The crude ligand was purified by column chromatography over neutral alumina, using a gradient of 100% CH_2_Cl_2_ to 5% CH_3_OH in CH_2_Cl_2_, to give a yellow sticky oil. Trituration of the oil with hexane gave the protected ligand **3** as a yellow sticky solid (47 mg, 96%). ^1^H NMR (500 MHz, CDCl_3_, Supplementary Fig. 3): δ 3.63 (quint, *J* = 6 Hz, 2 H), 3.32 (d, *J* = 6 Hz, 4 H), 3.24 (quint, *J* = 7 Hz, 4 H), 3.10 (q, *J* = 7 Hz, 4 H), 3.02 (br s, 4 H), 2.72 (br s, 4 H), 2.21 (t, *J* = 7 Hz, 6 H), 1.63 (quint, *J* = 8 Hz, 4 H), 1.43 (d, *J* = 7, 29 H). ^13^C NMR (125.7 MHz, CDCl_3_, Supplementary Fig. 4): 173.5, 172.7, 170.4, 170.0, 83.0, 82.6, 82.0, 56.5, 55.6, 54.1, 53.4, 51.2, 50.2, 42.4, 35.9, 35.5, 28.5, 28.1, 28.0, 27.9, 22.8, 18.6, 17.3, 12.4. HRMS (*m/z*): [M + H]^+^ calcd for C_33_H_63_N_8_O_7_, 683.4814; found 683.4834 (Supplementary Fig. 5).

*2,2′,2″-(10-(2-(5-azidopentanamido)ethyl)-1,4,7,10-tetraazacyclododecane-1,4,7-triyl)triacetic acid* (Fig. 1, 1). Trifluoroacetic acid (TFA, 0.6 mL) was added dropwise over 10 min to a stirred solution of tri-*tert-butyl* 2,2′,2′′-(10-(2-(5-azidopentanamido)ethyl)-1,4,7,10-tetraazacyclododecane-1,4,7-triyl)triacetate (Fig. 1, 3, 14 mg, 0.021 mmol)) in CH_2_Cl_2_ (1 mL) dropwise at 0 °C. The reaction mixture was warmed to RT and stirred at RT for 24 h. The solvent was removed under reduced pressure. Methanol (8 mL) was added to the residue and evaporated off to eliminate excess TFA. This process was repeated ten times to yield DOTA-Et-pentanamidoazide (**1**, 10.5 mg, quantitative). ^1^H NMR (500 MHz, CD_3_OD, Supplementary Fig. 6): δ 4.08 (br s, 2 H), 3.90 (s, 2 H), 3.64 (t, *J* = 6 Hz, 2 H), 3.58–3.53 (m, 4 H), 3.43–3.37 (m, 6 H), 3.23 (br s, 4 H), 3.14 (d, *J* = 7 Hz, 2 H), 3.05 (s, 2 H), 3.01 (br s, 2 H), 2.19 (s, 2 H), 1.59 (s, 2 H), 1.52–1.43 (m, 2 H), 1.28 (s, 2 H). ^13^C NMR (125.7 MHz, CD_3_OD, Supplementary Fig. 7): 176.8, 176.7, 162.8, 162.5, 119.2 (q, *J* = 291 Hz), 55.9, 55.6, 53.9, 52.2, 43.9, 36.2, 29.5, 28.6, 23.9, 18.8, 17.3, 13.2. HRMS (*m/z*): [M + H]^+^ calcd for C_21_H_39_N_8_O_7_, 515.2936; found 515.2914 (Supplementary Fig. 8).

*Tb-azide*. The ligand 2,2′,2′′-(10-(2-(5-azidopentanamido)ethyl)-1,4,7,10-tetraazacyclododecane-1,4,7-triyl)triacetic acid (Fig. 1, 1, 5.5 mg, 0.011 mmol) was dissolved in a CH_3_OH and H_2_O (1:1) mixture (15 mL) and the pH was adjusted to 7 with NaOH (aq.). TbCl_3_∙6H_2_O (4.0 mg, 0.011 mmol) was added to the reaction mixture and the pH was again adjusted to 7 with NaOH (aq.). The reaction mixture was stirred at 70 °C for 72 h. The pH was monitored periodically and adjusted to 7 with NaOH (aq.) as needed. The solvent was removed under reduced pressure. The solid obtained was dissolved in water (5 mL). The solution was centrifuged to remove the unwanted solid. The solvent was removed under reduced pressure to yield Tb-azide (Fig. 1) as off-white solid (6.9 mg, 96%). HRMS (*m/z*): [M + Na]^+^ calcd for C_21_H_35_N_8_Na_1_O_7_Tb_1_, 693.1774; found 693.1788 (Supplementary Fig. 10). Further characterization with ^1^H NMR, ESI-MS and HPLC is shown as supplementary data (Supplementary Figs 9, 11, 12).

### Data Availability

The datasets generated and analyzed for the current study are available from the corresponding author by request.

## Electronic supplementary material

Supplementary Figures
