# Supplementary material for: Development of a Click-Chemistry Reagent Compatible with Mass Cytometry
Source: Sci Rep. 2018 Apr 27;8:6657. doi: 10.1038/s41598-018-25000-y (PMC5923286; doi:10.1038/s41598-018-25000-y)
Supplement: Supplementary file 1 — Supplementary Figures [file 41598_2018_25000_MOESM1_ESM.pdf]

Development of a Click-Chemistry Reagent Compatible with Mass Cytometry

Jessica Shaklee, Kriti Srivastava, Heather Brown, Edgar A. Arriaga, Valerie C. Pierre, Jop H. van Berlo

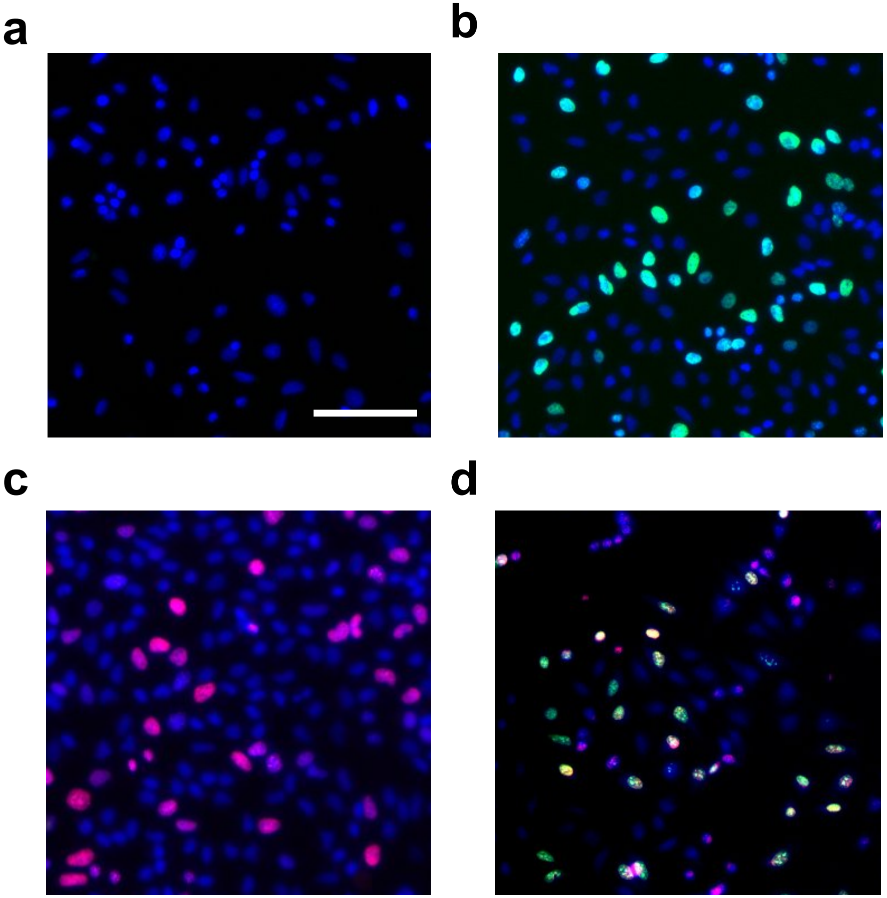

Supplementary Figure 1. Immunocytochemistry of BrdU and EdU incorporation
(**a**) Negative control stained for BrdU (red) and EdU (green) and DAPI (blue). No nuclei show red or green staining, showing specificity of staining. (**b**) BrdU staining of HeLa cells (green). Cells were treated with BrdU for 4h and stained with BrdU antibody (green) and DAPI (blue). (**c**) EdU staining of HeLa cells (red). Cells were treated with EdU for 4h and stained with rhodamine conjugated azide using click chemistry (red) and DAPI (blue). (**d**) HeLa cells treated with EdU for 4h followed by BrdU for 4h, stained for BrdU (green) and EdU (red) and DAPI (blue). Bar in (a) is 50μm.

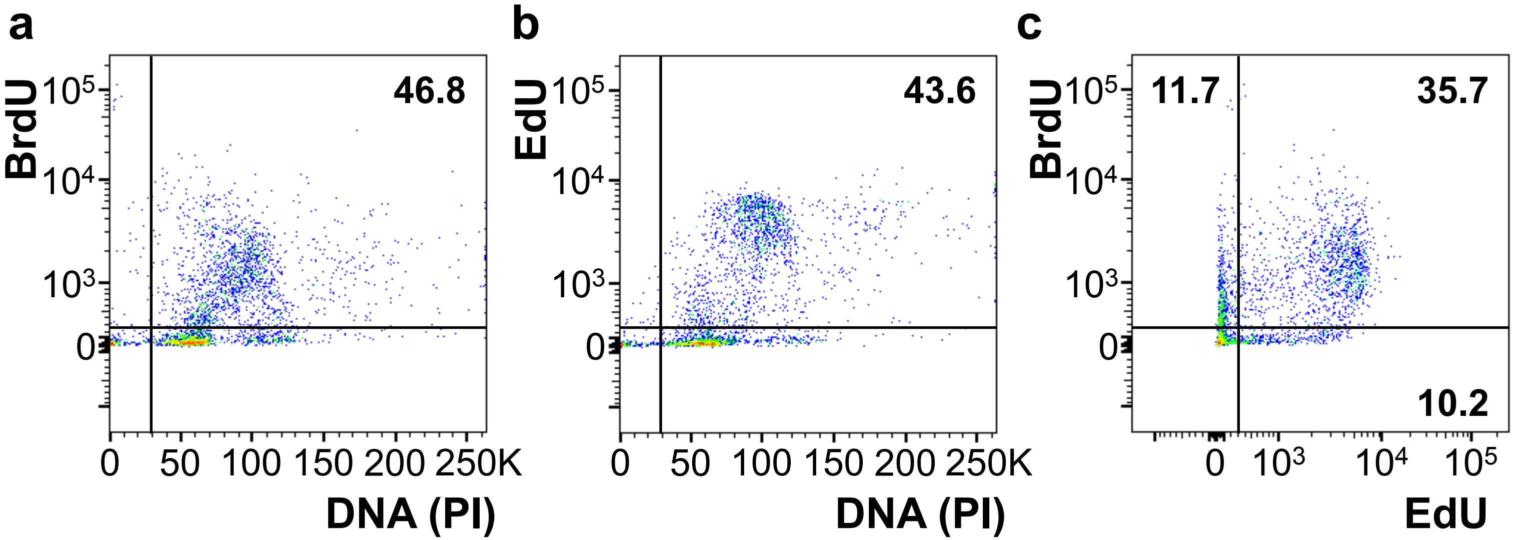

Supplementary Figure 2. Flow cytometry detection of BrdU and EdU incorporation
(**a**) Flow cytometry plot of BrdU stained HeLa cells showing BrdU vs DNA (PI) staining. Number in upper right corner shows percentage of cells labeled with BrdU. (**b**) Flow cytometry plot of EdU stained HeLa cells showing EdU vs DNA (PI) staining. Number in upper right corner shows percentage of cells labeled with EdU. (**c**) Flow cytometry plot of EdU and BrdU stained HeLa cells showing BrdU vs EdU staining. Numbers show percentage of cells that are positive for EdU and/or BrdU.

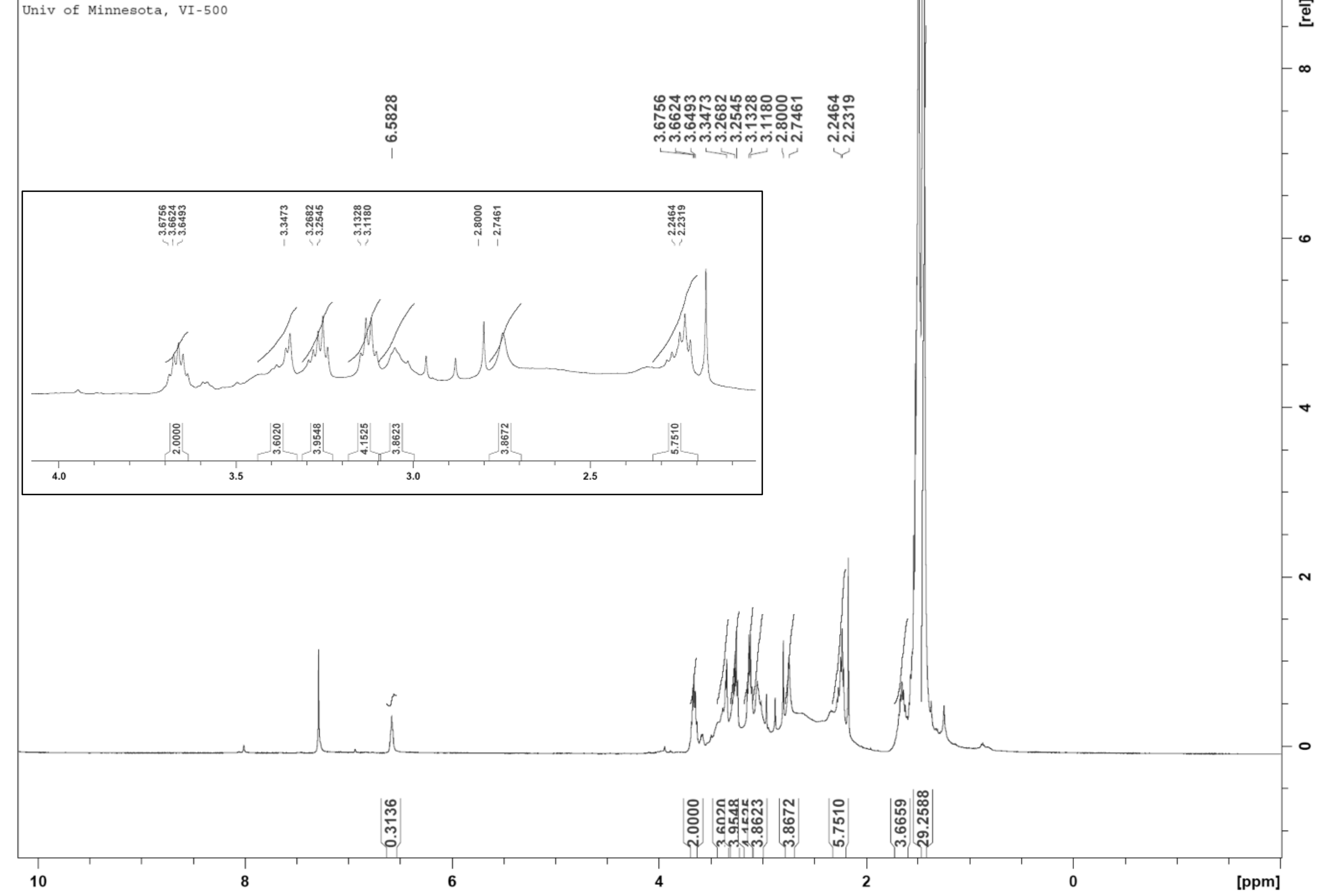

Supplementary Figure 3. ^{1}H NMR spectrum of DO3A-Et-pentanamidoazide (**3**) (expansion inset) ($CDCl_3$, 500 MHz).

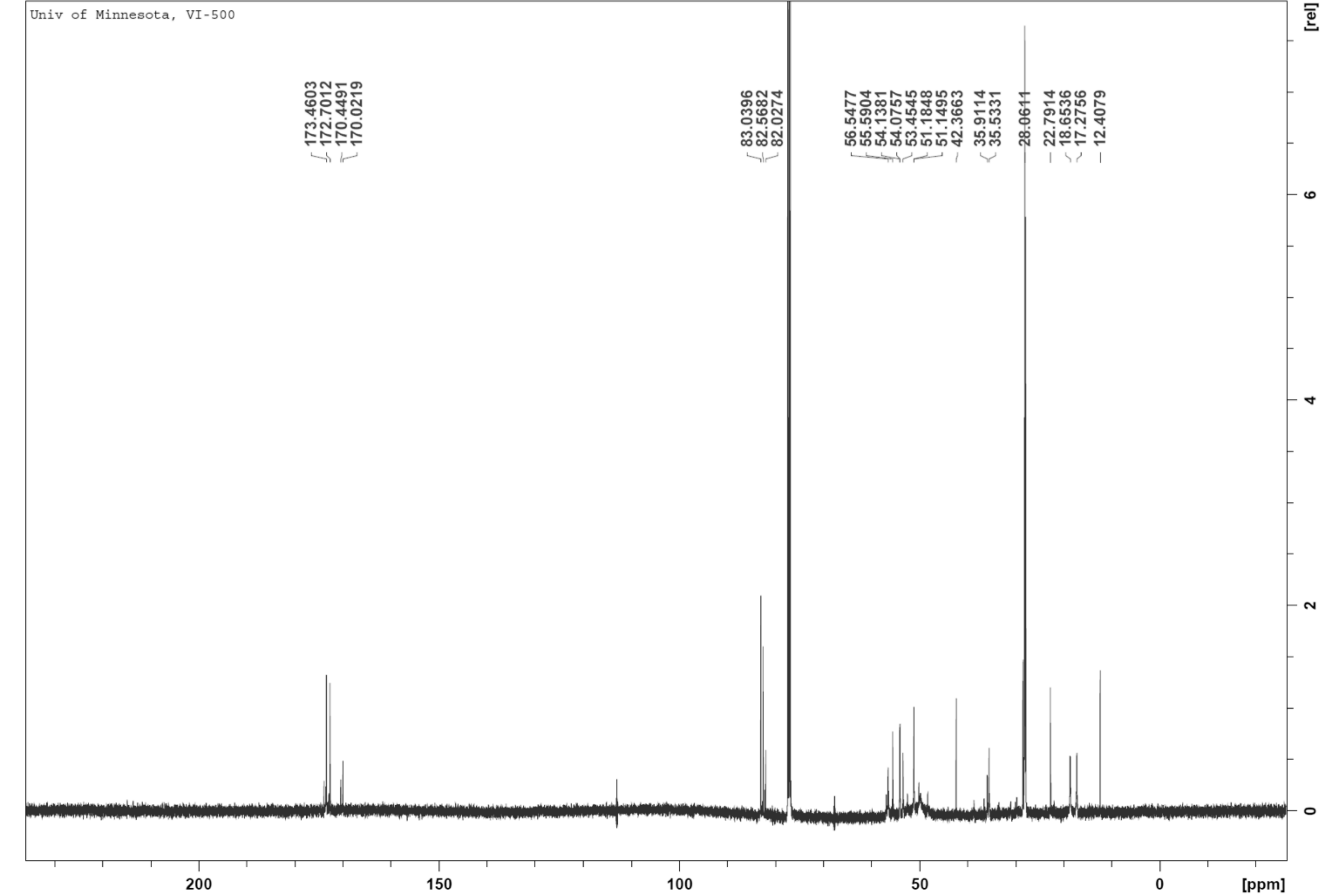

Supplementary Figure 4. ^{13}C NMR spectrum of DO3A-Et-pentanamidoazide (**3**) ($CDCl_3$, 500 MHz).

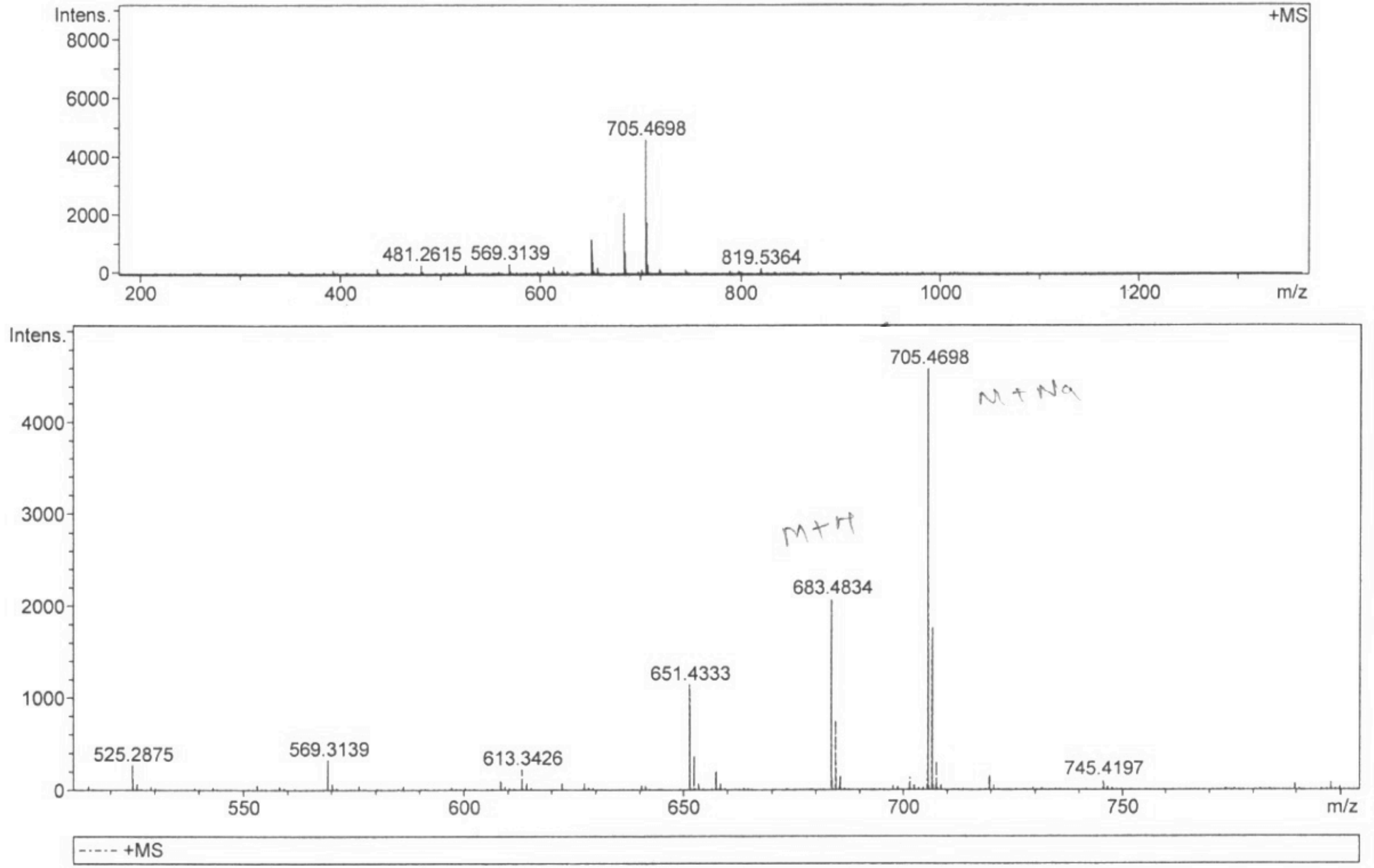

Sum Formula	Sigma	m/z	Err [ppm]	Mean Err [ppm]	Err [mDa]	rdb	N Rule	e⁻
C 33 H 63 N 8 O 7	0.023	683.4814	-2.88	-2.60	-1.97	6.50	ok	even

Supplementary Figure 5. HRMS spectrum of DO3A-Et-pentanamidoazide (**3**).

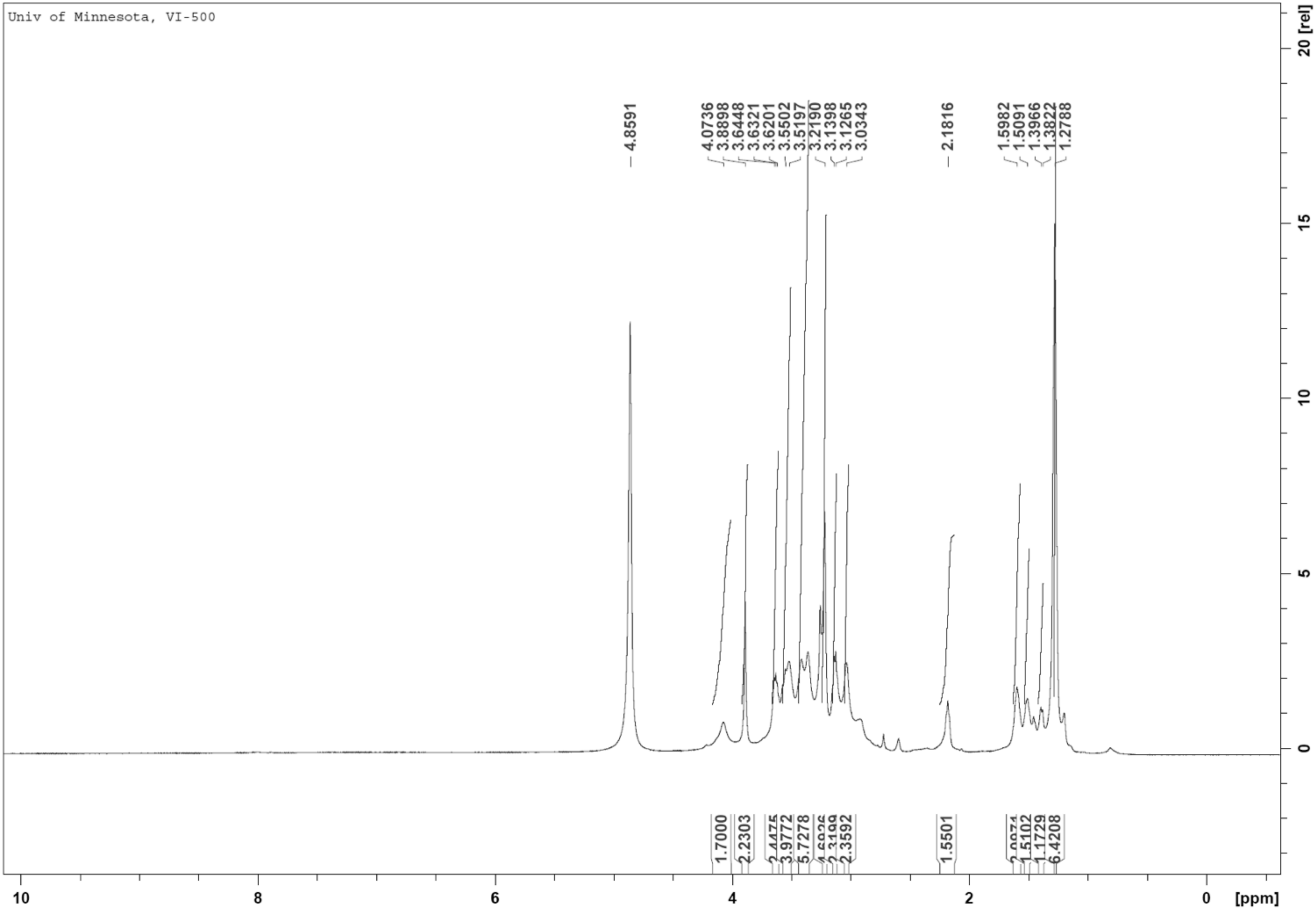

Supplementary Figure 6. ^{1}H NMR spectrum of DOTA-Et-pentanamidoazide (**1**) (CD_3OD, 500 MHz).

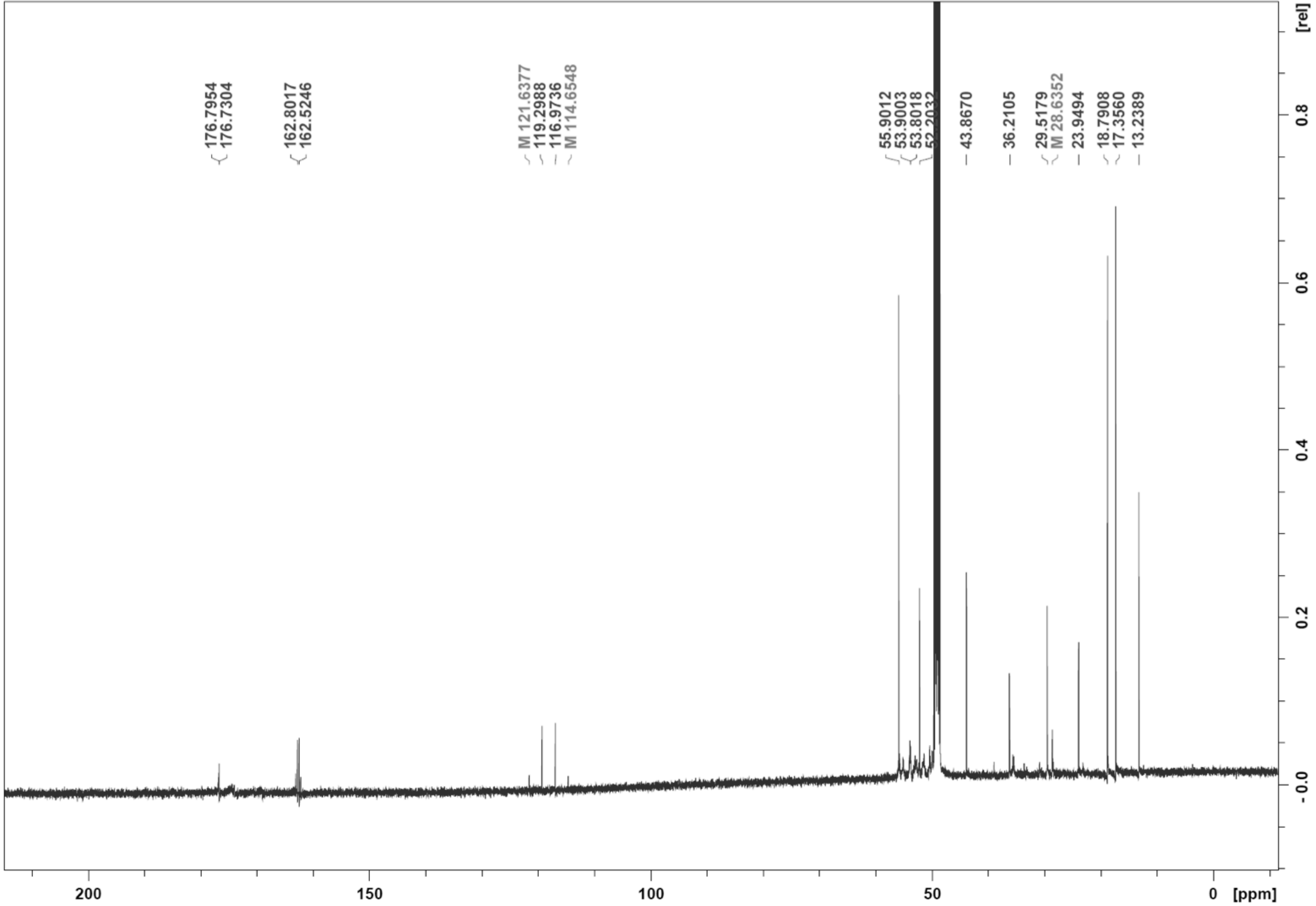

Supplementary Figure 7. ^{13}C NMR spectrum of DOTA-Et-pentanamidoazide (**1**) (CD_3OD, 500 MHz).

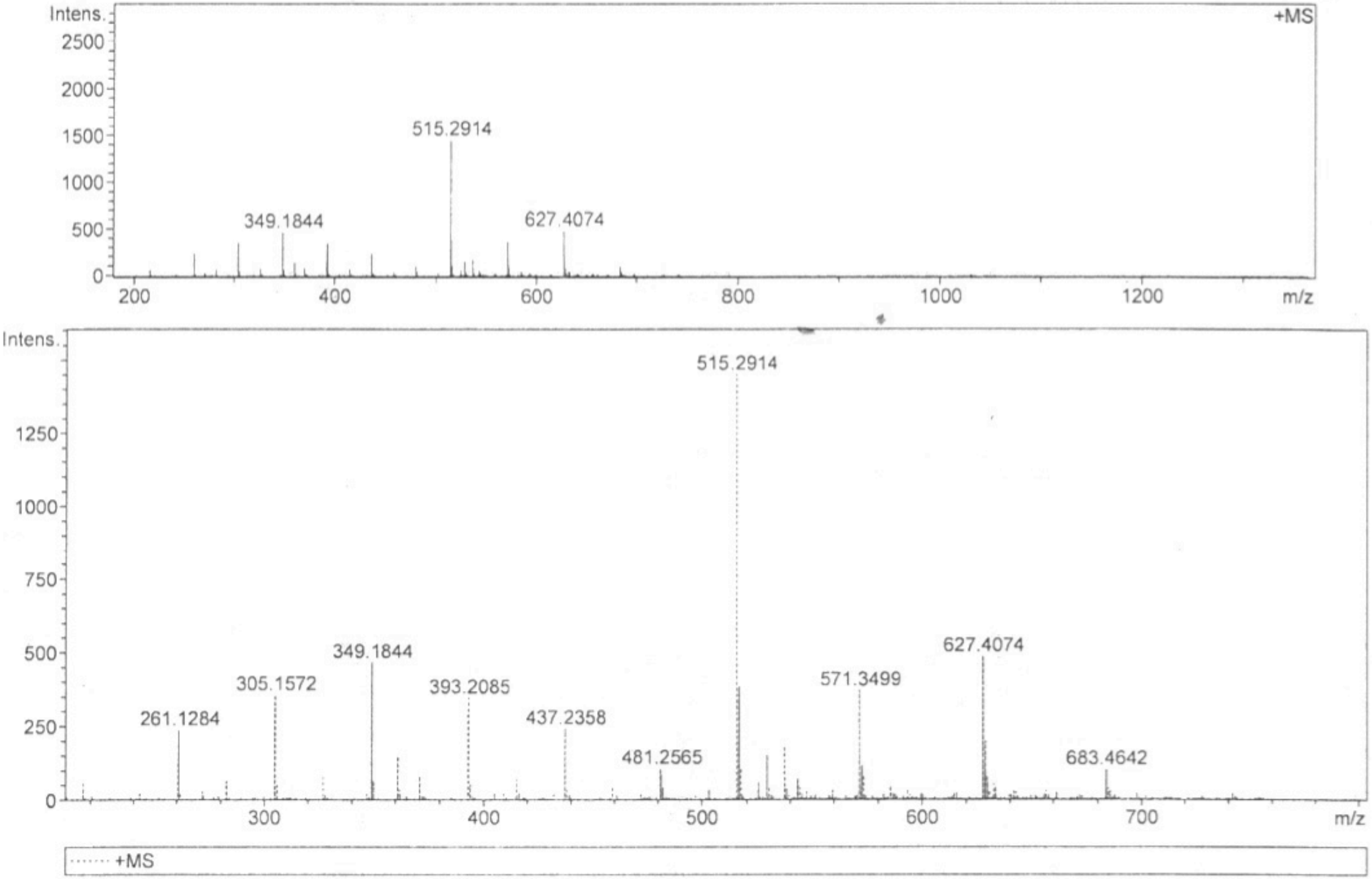

Sum Formula	Sigma	m/z	Err [ppm]	Mean Err [ppm]	Err [mDa]	rdb	N Rule	e⁻
C 21 H 39 N 8 O 7	0.015	515.2936	4.35	3.31	2.24	6.50	ok	even

Supplementary Figure 8. HRMS spectrum of DOTA-Et-pentanamidoazide (**1**).

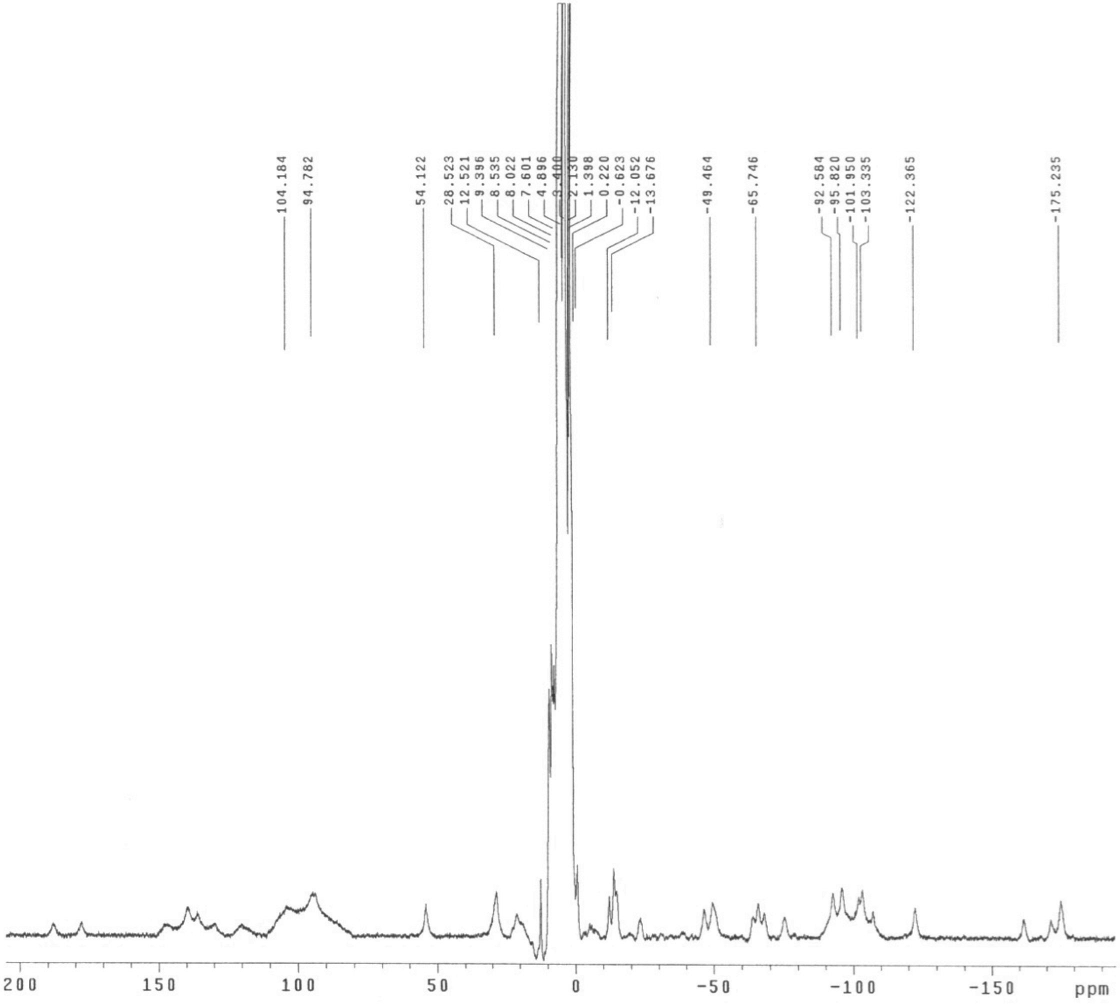

Supplementary Figure 9. ^{1}H NMR spectrum of Tb-azide (CD_3OD, 500 MHz).

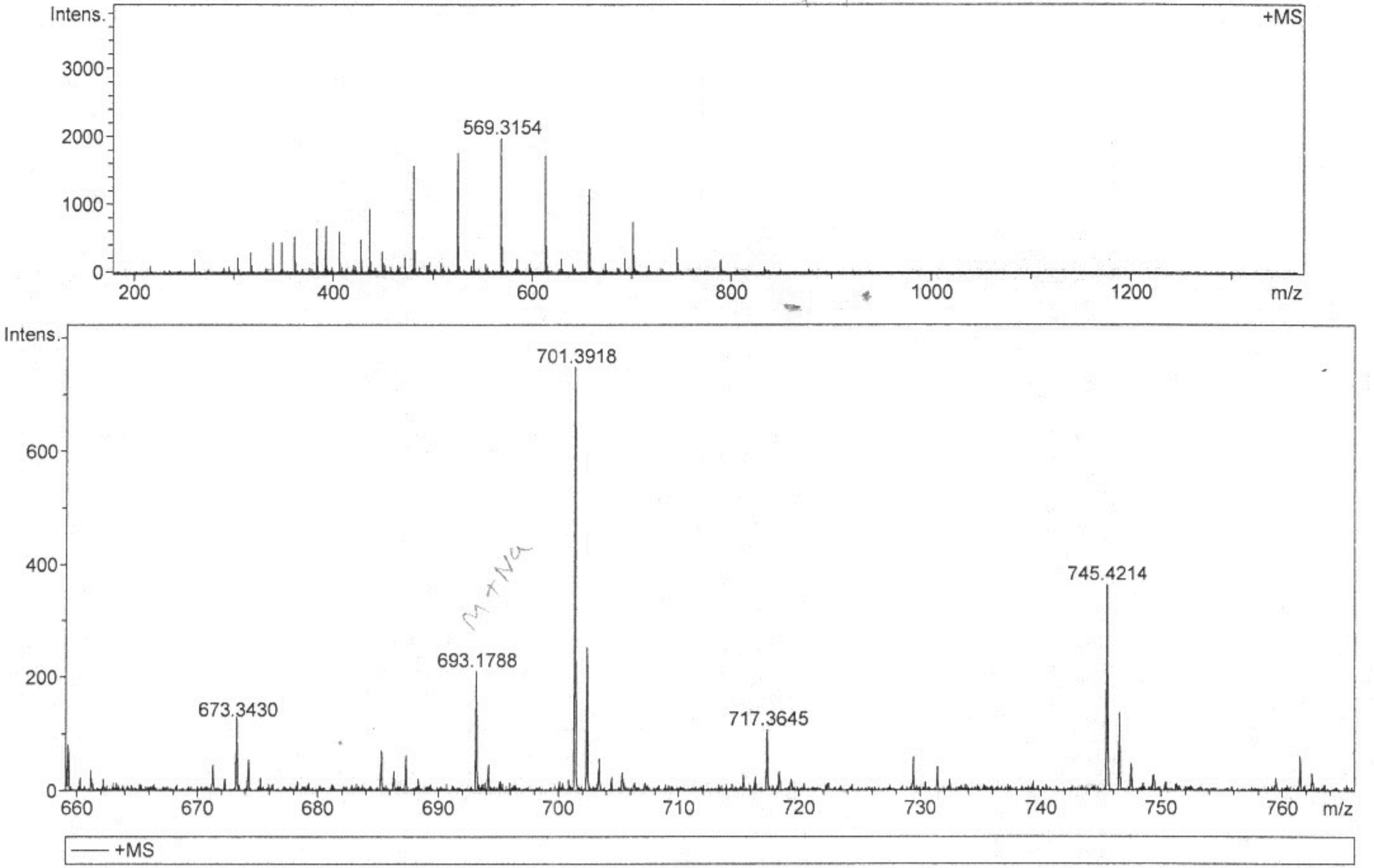

Sum Formula	Sigma	m/z	Err [ppm]	Mean Err [ppm]	Err [mDa]	rdb	N Rule	e⁻
C 21 H 35 N 8 Na 1 O 7 Tb 1	0.155	693.1774	-1.92	-1.90	-1.33	8.50	ok	even

Supplementary Figure 10. HRMS spectrum of Tb-azide.

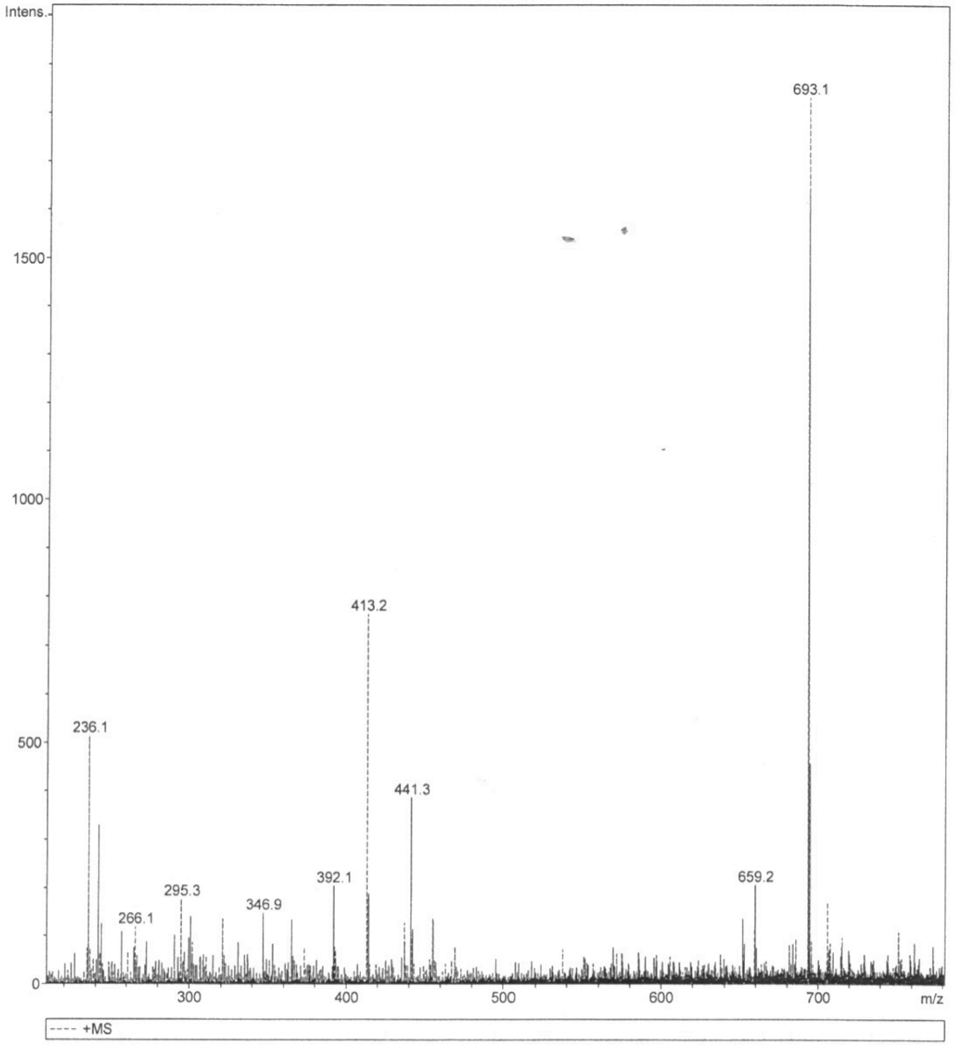

Supplementary Figure 11. ESI-MS spectrum of Tb-azide.

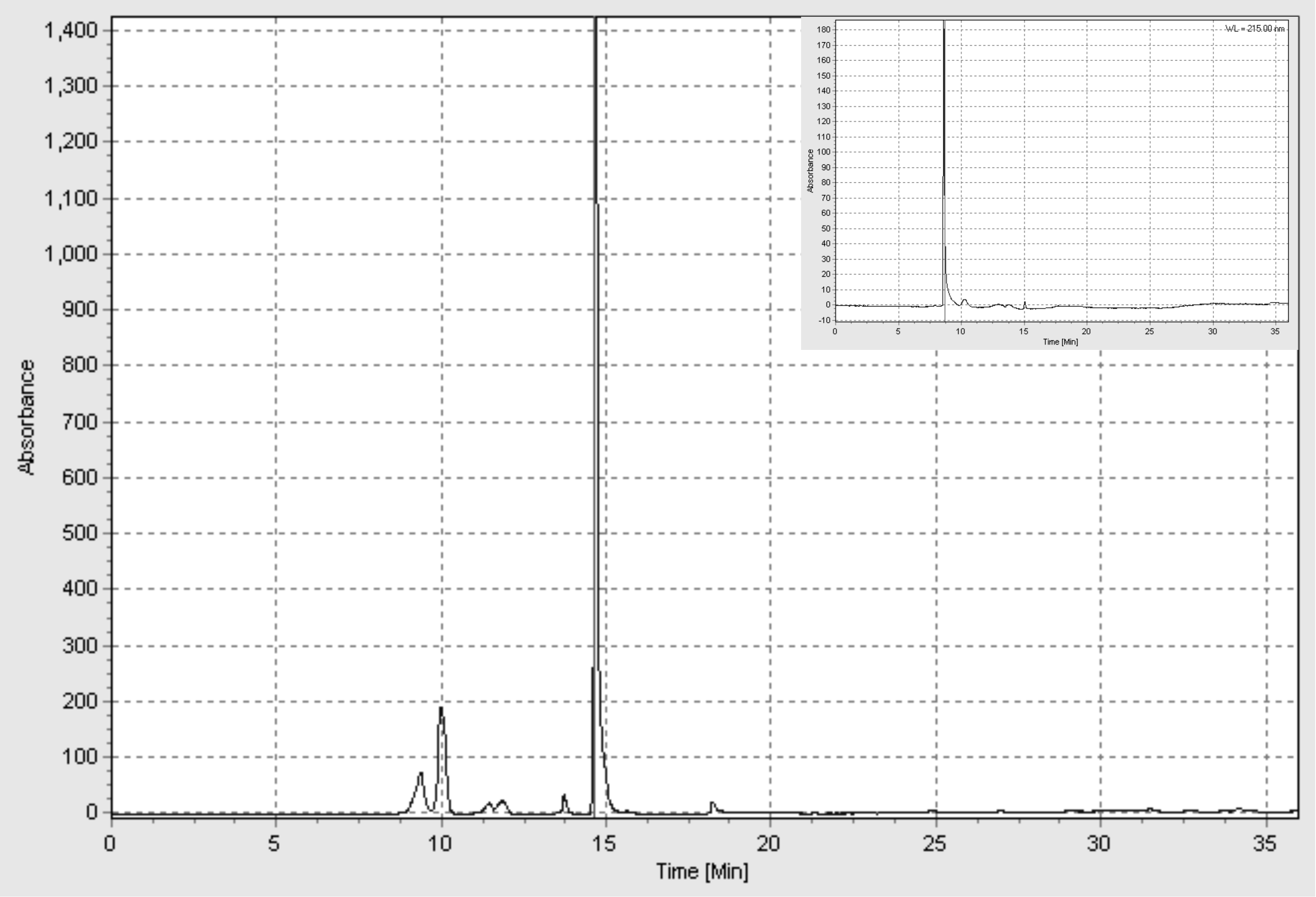

Supplementary Figure 12. HPLC trace of Tb-azide (background, inset). Experimental conditions: Varian Microsorb 300-5 C18 250 mm × 4.6 mm column, 1.0 mL/min flow rate, solvent gradient: 100% water to 85% water/ 15% CH_3CN in 2 min then to 100% CH_3CN in 23 min and holding at 100% CH_3CN until 26 min then back to 85% water/ 15% CH_3CN untill 35 min. At 14.65 min the product appeared and was identified by ESI MS.
